# Vascular normalization and immunotherapy: Spawning a virtuous cycle

**DOI:** 10.3389/fonc.2022.1002957

**Published:** 2022-10-06

**Authors:** Kumara Swamy

**Affiliations:** Radiation Oncology, Aster CMI, Bangalore, India

**Keywords:** normalization window, immunotherapy, anti-angiogenics, *in vivo* vaccine, SBRT, immunity cycle, HIF1-α, phagocytosis

## Abstract

Anti-angiogenics, radiotherapy (especially stereotactic body radiotherapy, SBRT)/chemotherapy, and immunotherapy form a critical trimodal approach in modern cancer therapy. The normalization window, however short, is the beachhead for the strategic initiation of a decipherable disruption of cancer cells. This opening can be the opportunity for designing controlled stepwise cancer cell death (CCD) and immunological augmentation. The next step is to induce immunogenic cell death (ICD) through chemotherapy/radiotherapy concurrently with the facilitation of professional phagocytosis. Immunotherapy at this stage, when interstitial pressure decreases considerably, leads to the improved perfusion of oxygen with solutes and improved immune-friendly pH and is additionally expected to open up the tumor microenvironment (TME) for a “flood” of tumor-infiltrating lymphocytes. Furthermore, there would be enhanced interaction in “hot” nodules and the incorporation of immune reaction in “cold” nodules. Simultaneously, the added adjuvant-assisted neoantigen–immune cell interaction will likely set in a virtuous cycle of CCD induction followed by tumor cell-specific antigenic reaction boosting CCD, in turn promoting the normalization of the vasculature, completing the loop. There should be a conscious concern to protect the extracellular matrix (ECM), which will nurture the long-term immunological cross-talk to discourage dormancy, which is as essential as obtaining a complete response in imaging. The caveat is that the available therapies should be appropriately ranked during the start of the treatment since the initial administration is the most opportune period. A fast-paced development in the nanomedicine field is likely to assist in all the steps enumerated.

## 1 Introduction

Immunotherapy (ImT) is the latest entrant to the armamentarium of cancer therapy, with much promise. However, it has the issue of low response rates, an average of around 12% across all tumor types, and the added secondary resistance over time. ImT also endangers a significant patient population at risk of substantial (financial and physical) toxicities, devoid of encouraging clinical benefits (1). With multitudes of mutations, phenotypes can vary from cell to cell, giving rise to varied cell populations within the cancer cell mass and the tumor microenvironment (TME), at different locations and temporally during the entire treatment period (2), which elicits a myriad of manifest and evolutionary latent pathways.

Invoking the anticancer response has been a fundamental approach in cancer immunotherapy, which is seeing a paradigm shift into a more specific immune activation process with fewer toxicities. There are two approaches to immune enhancement: one is passive ImT, where effector cells/molecules are utilized for a direct attack, and the second is where endogenous immune mechanisms are activated and are considered “active” immunotherapies. The encouraging results from anti-programmed cell death therapy and the improved outcomes in combination with radiotherapy (RT) and chemotherapy have opened avenues for normalizing specific defects in tumor immunity (3).

The primary objective of this article was to discuss the comprehensive targeting of the labyrinthine immunosuppressive aberrant tumor vasculature to increase the immunogenicity of, especially, the immune inactive “cold nodules” using ImT combinatorial therapies for recognizable clinical impacts.

## 2 Review of literature

### 2.1 Immunological classification of cancers

There are two crucial aspects for the categorization of ImT, irrespective of the histological type. One type comprises the immunologically hot and cold nodules. The “hot” characteristics include an immune cell-rich TME, a high number of cytotoxic T lymphocytes (CTLs) (i.e., CD8^+^ effector T cells), a high CD8/Treg (regulatory T cell) ratio, antigen-presenting cells (APCs), inflammatory M1-polarized macrophage infiltration, and immune stimulatory cytokine production [such as type I interferon (IFN)]. Cold nodules do not have these features. The second classification concerns the availability of tumor antigen due to a high tumor mutational burden or microsatellite instability and checkpoint expression on activated, antigen-exposed immune cells, along with programmed death-ligand 1 (PD-L1) on tumor cells, which can be “high” (positive) or “low” (negative) indicative of the probability of response to ImT (1).

### 2.2 Importance of normalized vasculature, oxygenation, and ImT

A long time back, it was recognized that one of the four fundamentals (four Rs) of fractionated RT is killing the oxygenated cells with initial fractions, relieving the interstitial pressure, facilitating the opening of the vessels, and activating the dormant cells in order to bring them under the ambit of sensitivity. Even the partially damaged cancer cells become susceptible to disintegration when the oxygen “fixes” them and prevents the process of repair, thus sensitizing these cells to any anticancer therapies (4).

The tumor vasculature comprises primarily hypoxia-induced distorted, malformed, and leaky inefficient vessels frantically formed to meet the exigency of the requirement of fast-proliferating cancer cells and manifested as “aberrant angiogenesis” (5, 6). Such a vasculature is a recipe for the development of resistance to the various cancer therapies coming in the way of uniform drug delivery, increased interstitial pressure with blood stasis, distorted lymphatic drainage, deteriorating immunological milieu with the presence of myeloid-derived suppressor cells (MDSCs), M2 tumor-associated macrophages (TAMs), and Tregs (5), which switch cancer cells into resistance phenotypes. Secondarily, tumor-promoting autophagy (resistance autophagy) and a damaged ECM with tumor-associated fibroblasts develop, conditioning the TME for increased local progression and metastases (5). In advanced malignancies, more often, cancer cells develop resistance after an initial response, and the hypoxia rebounds with consequent altered downstream changes and may no longer respond to further therapy. Preclinical studies have shown that, after the initial favorable response to anti-vascular endothelial growth factor/receptor (VEGF/VEGFR) treatment, increased vessel pruning later leads to tumor growth and metastases (5).

The success of ImT and the accumulation of T cells are multistep processes involving recruitment, expansion, and infiltration into the TME and the effective anticancer activity of immune cells such as CTLs (5). Generally, with the presently available therapies, the normalization of aberrant vessels remains for a specified period considered as the “normalization window” (NW) (7). It is advantageous to enhance or extend this period during ImT. The ImT itself has vessel-normalizing effects, as seen in preclinical models of breast cancer, although the kinetics of the same remains unexplored, and an increased risk of edema in brain tumors has been documented (6). The TME accumulation of both CD4^+^ and CD8^+^ cells confined to tumor areas with normalized vessels is established, although the overall number of T cells in the tumor mass remains unchanged (6), indicating the importance of normalization for ImT. However, this effect may not be long-lasting, and aberrant vasculature returns, leading to recurrence and progression (5).

The impact of the ImT depends significantly on certain factors that influence the intensity and width of the window of normalization and improved oxygenation, such as the dosage, its duration, and the sequence of anti-angiogenics (AAGs) in combination with ImT (a lower dose range of therapy can be more effective than the higher dose influenced by the tolerance of endothelial cells) and AAG interruptions in treatment (6, 7). The following points of the discussion revolve around the targeting normalization of the vasculature with improved oxygenation, which is a *sine qua non* for cancer control and is a foundation for an effective therapeutic strategy.

### 2.3 “Beyond” vascular normalization

Ideally, “normalization” of the vessel that is seen in the usual situation of AAG temporal action is not enough since its functional integrity may not be the most favorable given suboptimal pericyte coverage (initially deficient and later excessive), leading to the risk of hemorrhage, as seen in vascular endothelial growth factor 2 (VEGF2) blockade. Activation of angiopoietin-1 (Ang1) and the Tie2 (receptor tyrosine kinase) signaling pathway should kick in at the appropriate moment to promote vascular maturation (8). The other aspect being explored is that beyond normalization, i.e., inducing the formation of high endothelial cells in the vasculature (HEVs) for lymphocyte trafficking into the tumor, which may promote the formation of tertiary lymphoid structures in enhancing the effects of ImT. Anti-VEGFR2 antibodies, when combined with PD-L1 antibodies, induced the formation of HEVs and improved T-cell infiltration in breast and pancreatic cancer models (9). When coupled with a vascular targeting peptide ligand for the lymphotoxin beta receptor, normalization also induced HEVs in neuroendocrine tumors (9). Thirdly, drugs such as CU06-1004 further the normalization effects by advancing specific CD8^+^ T-cell activity and the antitumor cytokine, interferon gamma (IFN-γ), when combined with ImT. CU06-1004 is a small molecule and a dysfunctional endothelial blocker that sustains vascular stabilization by preventing endothelial loss and increases its efficacy through improved delivery of the AAG sunitinib (10).

### 2.4 Intransigent metabolic TME: Metabolic reprogramming

One significant hypoxia-induced intracellular change is the switch from oxidative phosphorylation to glycolysis, which leads to the accumulation of metabolic acids such as lactate and carbon dioxide (CO_2_) (11). To overcome this excessive intracellular acidosis, conversion of extracellular CO_2_ into hydrogen ion H^+^ (proton) and bicarbonate ion $HCO_{3}^{-}$ by carbonic anhydrases (CAs) takes place, and $HCO_{3}^{-}$ is absorbed intracellularly with H^+^ extracellular diffusion. The other mechanism is the transport of lactate across the plasma membrane through the induction of monocarboxylate transporters 1 and 4 (MCT1 and MCT4, respectively) (12). Thus, proton efflux through several types of acid transporters causes acidosis in the TME.

**Figure 1:**
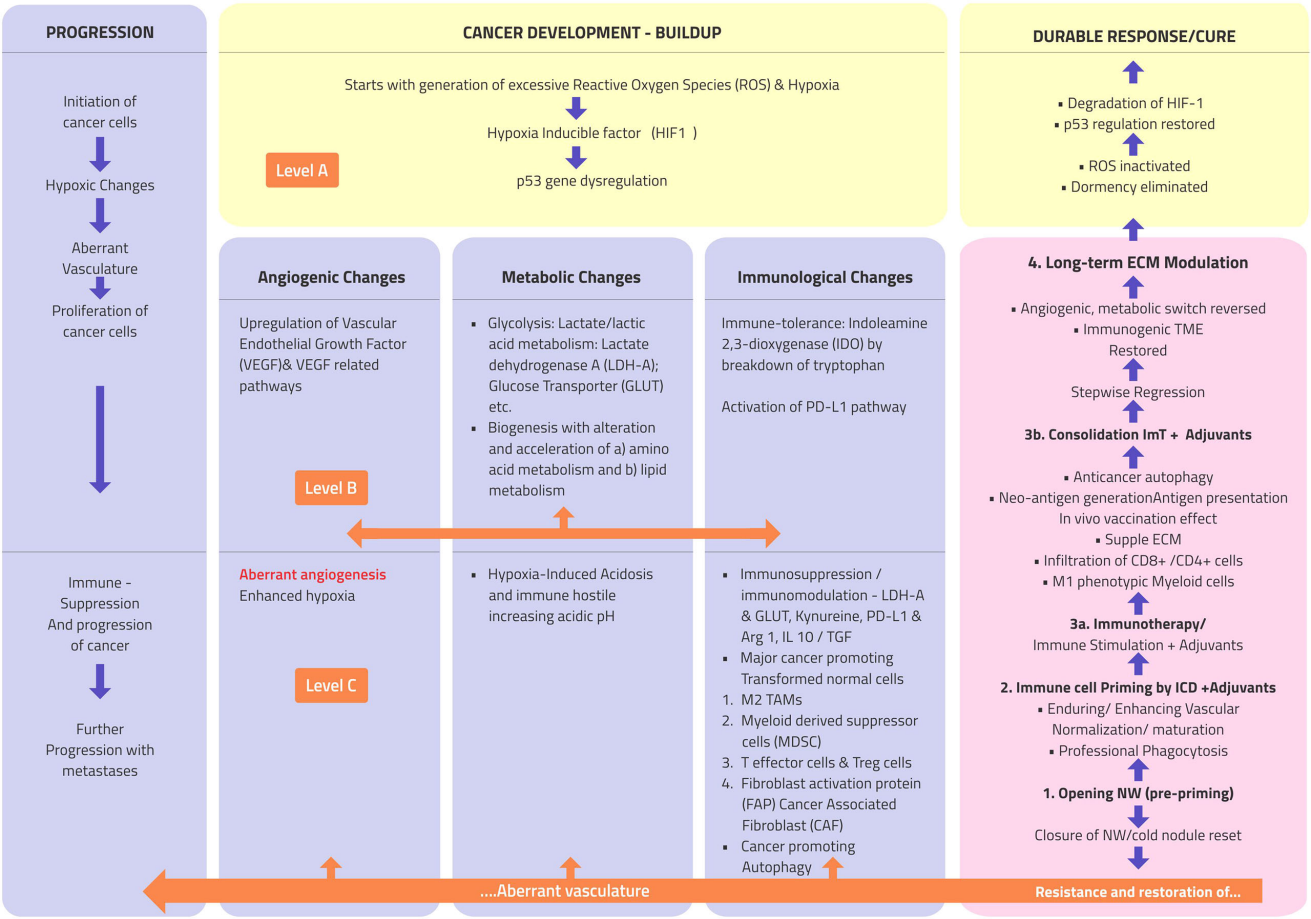
Atlas of the natural history, morphological and pathophysiological changes such as progression (*extreme left column*), and therapy regression/response (*extreme right column*) flow in cancer. *Level A*: three basic intracellular changes; *level B*: major downstream intracellular changes; and *level C*: major extracellular changes. *PD-L1*, programmed cell death-ligand 1; *TAMs*, tumor-associated macrophages; *IL-1*, interleukin 1; *Arg1*, arginase 1; *TGF-β*, transforming growth factor beta; *ROS*, reactive oxygen species; *ImT*, immunotherapy.

**Figure 1** presents a brief outline of the cancer conundrum. There are three primary changes at the intracellular level: the generation of excessive reactive oxygen species (ROS), the stabilization of hypoxia-inducible factor-1α (HIF-1α) due to marginal hypoxia, and the dysregulation of the *p53* gene (13). During its hypoxia-induced stabilization, HIF-1α, which typically disintegrates instantly, is accumulated in the cells, playing the maestro in coordinating all the subsequent evolution of cancer (**Figure 1**, level A). The secondary intracellular alterations are primarily three major events—angiogenic, metabolic, and immunological “switch”—influencing each other (**Figure 1**, level 2). These alterations initiate the self-propagating series of events consisting of shifting into a more straightforward method of burning energy using glycolysis for a surge in the biosynthesis of amino acids and lipid for proliferation and the development of immune tolerance by the breakdown of tryptophan to indoleamine 2,3-dioxygenase (IDO), escaping routine immune surveillance (13). These intracellular changes and the unchecked expansion of transformed cells lead to chaotic events in the extracellular compartment due to the seepage of cancer cell metabolite flux, further leading to an acidic pH and an immunosuppressive TME, along with the translation of normal protective ECM cells unwittingly facilitating the progression of cancer cells. With the increasing level of HIF-1α and progressive hypoxia in the cancer cells, signals for new blood vessel formation is initiated through the process of neoangiogenesis. Unable to keep up with the growing demand by the proliferating cancer cells, these vessels later become distorted, distinguishable as “aberrant" angiogenesis (**Figure 1**, level C) (13). All these changes make a fertile ground for the immunosuppressive milieu, giving rise to situations of inconsistent, unpredictable, and resistant responses to anticancer therapies.

While acute acidosis can induce apoptosis, chronic acidosis causes immunosuppression and is a conduit for multiple genomic mutations. The HIF-1α-mediated metabolic switch occurs not only in cancer cells but also in immune cells in the TME, promoting the immune inhibitory macrophage M2-like phenotype of TAMs and impairing innate immunity natural killer (NK) cell activity (13). Additionally, pH alterations act directly on the immune cells. An acidic pH resulting in the polarization of TAMs from the M1 to the M2 type, the increased presence of MDSCs and Tregs, inhibition of tumor-infiltrating lymphocytes (TILs), the accumulation of TAMs encouraging the anti-inflammatory cytokine interleukin 10 (IL-10), and transforming growth factor beta (TGF-β) leading to further lactate production set up a vicious cycle of immunosuppression in the TME (13).

Proton pump inhibitors have been shown to limit tumor expansion and restore the functionality of TILs. Ongoing studies have shown improvements in the increased alkalinity of the TME during ImT (13). Moreover, increased alkalinity has demonstrated restoration of the functionality of TILs in preclinical models (13). Hence, targeting metabolic reprogramming combined with ImT in the background of normalization of the vasculature would be rewarding.

### 2.5 Tracking normalization: Vasculature-guided ImT

Primarily, there are two approaches: imaging techniques when the measurable disease is present and liquid biopsy techniques when dealing with the microscopic residual disease. In the imaging field, magnetic resonance imaging (MRI), dynamic contrast-enhanced perfusion, and computed tomography (CT) studies have given indirect estimations of angiogenesis (14). New molecular imaging techniques such as positron emission tomography (PET) with paramagnetic nanoparticles, alpha-V beta-3 (a_V_β3) integrin receptor targeting, or sonography with gas-filled microbubbles directed against specific endothelial cell receptors can better estimate the effects of AAG therapy. Studies establishing the time interval of the normalization effects are vital in order to determine rational combinations of AAGs and to prevent the development of resistance (14).

The other avenue with a higher potential concerns the use of serum-based biomarkers to monitor tumor vascular NWs, which has the advantage of being able to be administered repeatedly and for a longer duration than that in animal studies. Estimates of serum markers like soluble VEGFR by-product (or soluble fms-like tyrosine kinase 1, sFlt1), Ang1/Ang2 (angiopoietin-2) ratio, hypoxia-regulated apelin, and vascular normalization induction factor thrombospondin-1 (TSP-1), among others, provide avenues for the monitoring of the vascular NW (15). The messenger RNA (mRNA) expression of apelin (an endogenous peptide ligand) has been demonstrated in preclinical studies to indicate a NW induced by bevacizumab. In addition, several microRNAs (miRNAs) are being evaluated (15), opening exciting possibilities for monitoring vessel normalization in clinical situations to facilitate an efficient synchronization of therapies.

*Highlight*: Presently, with the available therapies, normalization of the vasculature only lasts for a short duration, requiring innovative combinations for extension and enhancement. It is possible to track the vascular changes with modern imaging/molecular techniques, heralding the onset of vascular-guided ImT for the prompt evaluation of the adopted strategies.

### 2.6 Opening-up normalization window: Pre-priming for ImT

As shown in the literature, normalization of the vasculature with any therapy is temporary and time-bound (7). All treatments cause toxicities to a greater or a lesser extent, especially when administered repeatedly, and should be optimally used at the first opportunity before resistance develops. The most effective therapies require the simultaneous presence of oxygen for cancer cell death (CCD) and efficient delivery to the site and within the cancer mass when systemically administered. Theoretically, opening the NW of the vasculature (pre-priming) before initiating therapies that are known to induce significant CCD (priming) is broadly necessary for an avalanche of immunological action of ImT. Of all the known approaches, AAGs have been the most clinically examined group of drugs that have demonstrated to invoke this window of normalization to a significant extent. Studies have shown an improved effect when RT was given during the NW of AAGs, although the proper dose and sequencing are open for exploration (16). The effectiveness of the AAG drug class is attributed to this NW during the initial period of therapy, and continued administration leads to the excessive pruning of vessels, resulting in the closure of the window and exaggerated rebound hypoxia. Therefore, a matched combination with ImT is a valuable strategy for improving drug delivery and effectiveness, simultaneously reducing toxicity (6). Cediranib in glioblastoma multiforme consistently reduced tumor enhancement by 24 h as part of the initiation of the NW. The NW of AAGs was demonstrated to last at least 28 days, with decreased permeability that persisted even at day 112. With imaging, this normalization has been shown to have reversed after stopping, and normalization phenotypes were restored (7).

#### 2.6.1 Dose, duration, sequence, and timing

A study has shown that, in mice, treatment with a lower dose of 10–20 mg/kg (but not a higher dose range of 40 mg/kg) of the VEGFR-2-specific monoclonal antibody DC-101 in glioma xenografts increased vessel normalization, creating a time window for the synergistic effect for combined therapy with RT-induced ICD (5). The treatment accelerated the T helper type 1 (Th1) cell-mediated immune responses, including TAM polarization to an M1-like phenotype, increased CD4^+^ and CD8^+^ T-cell tumor infiltration, and reversed dendritic cell (DC) maturation defects. Additionally, ICD can be used for developing next-generation DC-based vaccines (5). The effect of AAGs causing the pruning of immature blood vessels, active pericyte recruitment initially normalizes the blood vessels starting as early as day 1, possibly lasting a few months in humans, is a “normalization window.” This window later ends up with the extensive pruning of blood vessels, resulting in alternative angiogenic pathways or blood vessel formation, such as vessel co-option (6). The other associated findings are that higher doses of AAGs caused excessive pruning with a shorter window, increased deposition in the ECM fertile ground for immunosuppressive and/or pro-tumor immune cells, and exacerbated hypoxia. When combined with chemoradiotherapy, bevacizumab, at a lower dose of <3.6 mg/kg, showed better survival than at a higher dose of 5 mg/kg in patients with glioblastoma (6). In a mouse model, after 7 days of anti-VEGF therapy, empty sleeves of basement membrane and pericytes survived, followed by endothelial sprouting to empty the basement membrane scaffold on stopping the drug within a day. Restoration of the vessel patency occurred with blood flow and repair of pericytes with a baseline phenotype by 7 days, which, notably, once again regressed as much on restart of the second treatment (17). In another study, during AAG drug holidays, due to toxicity, the normalization phenotype was restored the second time (7). Therefore, theoretically, an intermittent/cyclical NW can be established by re-challenging with the same or an alternative AAG.

The results of clinical studies targeting both Ang1 and Ang2 have been disappointing since the inhibition of pericyte-derived Ang1, which is an agonistic ligand of Tie2, might mitigate the benefits of vascular normalization by the blockade of Ang2 (6), making a case for Ang2 blockade cyclically and Ang1 accumulation in the temporal scale, keeping in mind that Ang2 is required for the initiation of angiogenesis and Ang1 for the later maturation of vessels. The bleeding complications reported with AAGs could probably be obviated by targeting the accumulation of Ang1.

*Highlight*: NW is dose-dependent and is initiated almost immediately after starting AAG therapy, and the second window manifests on restarting after a period of drug holidays (7). A trimodal approach of RT/chemotherapy, AAG therapy, and ImT synchronized in different timelines is a promising therapeutic strategy (16).

### 2.7 Cancer cell damage–death–disintegration: Priming for ImT

#### 2.7.1 The most powerful way of normalizing the vasculature and sustaining it is by inducing effective cancer cell death before the resistant mutations develop

Oxygen perfusion improves with the significant disintegration of cancer cells and reduced interstitial pressure; TILs flood the TME and organize the turf for ImT. Chemotherapy and RT are excellent methods that can be optimized to realize this need. There is more than one way cancer cells die, with each impacting the effectiveness of ImT in a varied mechanism, which needs to be harnessed. There is a need for insights into the interaction between RT, chemotherapy, AAG therapy, and ImT. The opportunities and challenges are in dose scheduling and timing (16). However, the basis for all these strategies is normalizing the tumor vasculature as the target, impacting reoxygenation and TIL infiltration. Recently, the abscopal effect has been garnering attention, with RT linked to the distant effects on the immune system (16).

#### 2.7.2 Mechanisms of CCD

There are several mechanisms of cell death documented: apoptosis, necrosis, autophagy, senescence, and ICD. The hallmarks of ICD include endoplasmic reticulum (ER) stress response by a defined emission of danger signals through surface calreticulin and secreted adenosine triphosphate (ATP), a potential pathway for “*in situ*” vaccines (5). All types of CCD, except necrosis, have been demonstrated to be capable of being immunogenic (autophagy can be immunosuppressive or immunogenic depending on the TME). However, necrosis contributes to chronic inflammation, creates a pro-tumorigenic immunosuppressive TME (18), and acts as a nidus for highly resistant anoxic tumor cells, especially in the wall of the necrotic cavity. Indeed, the conventional radiation principle dictates a dose delivery with nil/minimal necrotic component of cell death, dating back decades. Senescence has significance in the sub-vascular disruptive dose of cytotoxic therapy, where normal tissue (especially endothelial cells) withstands acute mitotic catastrophe, but not oxidative stress and degeneration over the long term (18). The phenomenon of senescence indicates the importance of moderation of the cytotoxic dose, where not only should the schedule be non-disruptive to the vasculature immediately but should also not be taken to the level of inducing potential significant senescence years later.

#### 2.7.3 RT and ImT: Cellular, vascular, and immunological effects

Broadly, there are two primary approaches to RT. Over the last 100 years, a gross disease with surrounding microscopic clusters of infiltration into the surrounding tissue and regional lymph nodes has been treated, exploiting improved oxygenation with gradual cell killing over 6–8 weeks. Moreover, this approach encompasses a significant amount of normal tissue; hence, the dose of 1.8–2 Gy per fraction evolved since normal tissue tolerance was the limiting factor. With improved technology, intensity-modulated radiotherapy (IMRT), image-guided radiotherapy (IGRT), and the consequent sparing of more normal tissue, a hypofractionated RT around 3 Gy per fraction is now practiced in certain situations. Some preclinical studies have suggested the superiority of hypofractionated radiation, while others found that conventional fractionation induces a more significant immune response (19).

With the evolution of the technique of stereotactic RT [stereotactic radiosurgery (SRS) and stereotactic body radiotherapy (SBRT)] with a dose per fraction of >5 Gy, it is designed to treat only gross disease with a rind of normal tissue. SBRT is unsuitable for the inclusion of a larger volume of normal tissue, such as in conventional fractionation; hence, both are not mutually exclusive. SBRT has a different mechanism of action, primarily through ICD, compared to conventional RT, which acts mainly by apoptosis. Conventional RT is considered immunosuppressive since the treatment is prolonged (20), which inhibits initiation and sustenance of innate immunological response. SBRT, with the ICD mechanism, has kindled the interest in invoking both local and distant (abscopal) immunological responses, hence may be ideal for combining with ImT appropriately.

When the dose per fraction approaches above 10 Gy, it is expected to cause tumor vasculature endothelial damage and vascular disruption, which reduces blood flow and perfusion, interfering with the delivery of ImT and the consequences of increased hypoxia. The majority of studies showed that it is vital to use the vascular non-disruptive dose of SBRT of 5–10 Gy (20) unless the endothelium is protected by genetic modulation (21) or the adoption of dual-recombinase technology (DRT), enunciated by Moding et al. (22), for maximizing the local and systemic (abscopal) immunological effects.

RT has multiple effects beyond the simple lysis of cancer cells on stromal elements such as fibroblasts, connective tissue, vasculature, and immune cells (20). If the dose per fraction nears a disruptive vasculature level, then stromal damage will induce progressive immunosuppressive fibrotic changes and a disproportionate increase in long-term side effects. A single dose of 12 Gy triggered significant TGF-β related to immune-hostile TME disruption, whereas release was minor with an SBRT dose of 6 Gy (20). The fractionated and not the single dose (20 Gy) of RT, combined with an anti-cytotoxic T-lymphocyte-associated antigen 4 (CTLA-4) antibody, produced an abscopal effect in a murine model (23). Furthermore, a dose per fraction range of 12–18 Gy induced *Trex1* (gene) exonuclease and attenuated any immunogenic effects compared to the lower doses that helped with IFN-γ production, DC recruitment, and immune cell priming (1). In the animal model, when the dose per fraction approached 50 Gy, it became palliative with only short-term tumor control (22). In general, doses above 10 Gy can cause extensive endothelial damage with the reduced vascular flow, impairing effector T-cell recruitment, thus exacerbating the hypoxia-driven immune-hostile TME. Regimens using <10 Gy per fraction might induce sufficient CCD without worsening the hypoxia (20) to initiate local and abscopal immune cascade.

#### 2.7.4 Timing of RT with ImT

Most studies have indicated that ImT administered after local therapy priming may be the most effective way of initiating, sustaining immunity, and overcoming T-cell exhaustion. This strategy requires validation by the ongoing studies NCT03738228 and NCT03799744 (1). The mechanisms and limitations of RT are discussed in detail above, and it can be an affordable and effective partner for ImT in effectively targeting the vasculature and the TME. One immunological classification of tumors is lymphocyte-rich “hot” tumors that respond well to ImT, while the majority are “cold” tumors indicative of a low overall response rate. Data on how often RT can help ImT convert the cold into the hot type, either locally or abscopal, are limited (20). In the combination of RT and ImT, RT has been hypothesized to possess actions complementary to those of immune checkpoint blockade, and by reciprocal action, ImT may also have radiosensitizing effects through vascular normalization (19). Regarding the timing, most studies have indicated that ImT within a week of SBRT is the most immunogenic (24).

#### 2.7.5 Further combinations

The reactive oxygen species (ROS)-dependent radiation effect on DNA requires the presence of oxygen, and a well-perfused tumor tissue is essential for the optimal action of RT. Daily fractions of up to 2 Gy are known to improve the tumor vasculature and tissue perfusion in addition to the growth of new blood vessels. However, after the initial response of radiosensitive cells, several negative phenomena occur, which come in the way of eliminating the remaining cancer cells and, hence, need to be addressed in clinical trials. These include: a) the repopulation after day 21 of resting/resistant phenotypic cells, accelerating the proliferation of cancer cells (4); b) vascular rebound due to VEGF/placental growth factor (PIGF)-initiated angiogenesis and the triggering of vasculogenesis (a form of VEGF-independent angiogenesis) due to the influx of endothelial progenitor cells from the bone marrow (16) (bone marrow-derived precursor endothelial cells, BMDCs); c) the recruitment of MDSCs inhibiting the activity of CD8^+^ T cells and DCs. Studies have shown the rationale of combining RT with immunotherapies that target MDSC and/or M2 macrophage recruitment and polarization to accelerate the antitumor immune response (25). ImT normalized the vasculature through the accumulation of IFN-γ-producing CD8^+^ cells and through Th1 cells (19). Lastly, d) the “exhaustion” of RT recruiting and activating CD8^+^ T cells, expressed as increased immune checkpoint receptors such as programmed cell death protein 1 (PD-1), with ensuing PD-L1-dependent resistance. This RT-induced increase in PD-L1 can be exploited by combining it with ImT. However, resistance can still develop, as shown in studies where IFN-γ and type I IFNs promote resistance to RT when combined with anti-CTLA-4 treatment in a PD-L1-independent manner (25).

#### 2.7.6 Taming the “dragon”

When the dose is >5 Gy per fraction, ICD releases damage-associated molecular patterns (DAMPs), including the translocation of the ER chaperone calreticulin and secreted ATP, which recruits and activates DCs and phagocytic macrophages. However, adenosine, the catabolic product of ATP, rapidly accumulates, suppressing DCs and immune cells, simultaneously promoting Tregs and TAMs to the M2 phenotype through TGF-β. When combined with RT, a newly developed bifunctional fusion protein can block both the PD-L1 and TGF-β pathways (25). The other important consequence of an SBRT dose schedule is that it may trigger two waves of BMDC influx, one within 3–5 days after exposure and a second delayed wave after about 2 weeks, associated with hypoxia. According to a study, this was dose-dependent since 8 Gy increased the BMDC influx to a minor degree, which more than doubled with 15 Gy per fraction. An inhibitor of the stromal cell-derived factor 1 (SDF-1)/chemokine receptor 4 (CXCR4) axis blocked this surge when given immediately after radiation (20).

*Highlight*: Most studies have shown that the SBRT dose per fraction of 5–10 Gy causes significant cell lysis and is most immunogenic when given just before ImT (20). With this dosing schedule, SBRT aims to normalize the vasculature early and initiate immune cell priming before local immunosuppression comes into the picture.

#### 2.7.7 Chemotherapy-induced ICD: Cellular, vascular, and immunological effects

Cancer cells can be driven to ICD by chemotherapeutics, especially drugs from the anthracycline family, oxaliplatin, bortezomib, cyclophosphamide, mitoxantrone, and oncolytic virotherapy, among others. Taxanes cause ICD to a limited extent. On the contrary, melphalan and cisplatin may be unable to generate ICD since they cannot cause ER stress. Standard chemotherapy is decided on indications, not the ability to induce ICD. Nanoformulations are evolving, which can improve the delivery of ICD-inducing chemotherapies. The translational field is focused on combining chemotherapy-induced ICD (CT-ICD) with ImT, especially the inclusion of at least one CT-ICD inducer in any given combination. The ineffectiveness of CT-ICD does not mitigate the activity of ImT (CT-ICD is an add-on benefit) (26), probably compensated by the chemotherapy-induced cell killing with ensuing improved oxygenation for the remaining cells.

Another advantage of chemotherapy is taking care of possible increased metastases with normalization. Since chemotherapy facilitates ICD-induced T-cell priming and remodels the immunosuppressive TME, it should be expected to be administered before ImT in order to maximize efficacy (27).

### 2.8 Neoantigens, immune priming, immune adjuvants, and “switching” on *in vivo* vaccine cycle

The success of vaccination against infective diseases is cornered by the use of immune adjuvants, which by themselves are not effective against microorganisms. The immune evasiveness of cancer cells is the fundamental cause of cancer initiation and progression. Data on the drugs that can enhance the interaction between cancer neoantigens and ImT are available. The systemic use of these adjuvants along with ImT will facilitate the identification of cancer cells and can act as an active *in vivo* therapeutic vaccine (28, 29).

Unlike a molecularly defined *in vitro* vaccine, an *in vivo* vaccine has downsides, e.g., the neoantigens released by such strategies will be “drowned” by the large amount of non-mutant peptides released simultaneously, and the control over the maturation signals received by APCs is limited. However, the advantages include the relative ease of clinical development (28) and, most critically, keeping up dynamically with the antigenic heterogeneity and the evolution of every resistant mutation during the therapy *in vivo*.

One method used is to stimulate natural immunity through Toll-like receptors (TLRs) and other innate recognition pathways. Well-timed immune adjuvants (which might be the oldest ImT on their own) have the potential to enhance the efficacy of ImT and RT by creating an active endogenous vaccine. However, the inflammatory actions of adjuvants can also lead to adaptive immunosuppression, which requires the inhibition of IDO. TLR9 is expressed in B cells, DCs, macrophages, and monocytes, and it recognizes unmethylated CpG oligonucleotide sequences. The CpG oligonucleotide, known for local action, when systemically administered, is the most effective when compared with any other TLR ligand inducing the activation and maturation of DCs, NK cells, and cytotoxic T lymphocytes and the differentiation of B cells into antibody-secreting plasma cells (29). SBRT combined with FMS-like tyrosine kinase 3 ligand (FLT3L), which mobilizes DCs during inflammation, is being tested in a non-small cell lung cancer phase II trial (30).

#### 2.8.1 Immunogenic antigen generation and priming

In a three-arm phase II Neo-CheckRay study, with different combinations, three priming strategies were adopted—chemotherapy, radiation therapy to the primary luminal breast cancer, and blockade of the adenosine pathway—the results of which are awaited (31). Immune adjuvants can stimulate immature DCs in capturing tumor-associated antigens, followed by maturation, lymph node migration, and the priming of naive T cells, thus increasing the probability of specific neoantigen presentation (2). A multi-arm immunotherapy study (NCT03804944) aims to discover the utility of CDX301, a recombinant human FLT3L, as an *in vivo* vaccination strategy (32).

#### 2.8.2 Nanomedicines

Nanomedicines improve the delivery of antigens and adjuvants to lymphoid tissue. Nanomedicines of 10- to 100-nm diameter improve the controlled release, retention, efficiency, and antigen cross-presentation of vaccines in draining lymph nodes, promoting the development of CD4^+^ T helper antitumor phenotypes. Polymers and lipids can themselves be designed to act as adjuvants stimulating the stimulator of interferon genes (STING) signaling pathway. Nanomedicines can combine multiple immunotherapies, chemotherapy, photothermal and photodynamic therapies, radiosensitizers, RNA interference-based immunomodulators, and other types of vaccines, all loaded with adjuvants. RT, through its normalization of the TME, improves the distribution of nanomedicines in mouse models (2). Nanomedicines, therefore, open up the possibility of improving the delivery of drugs, normalizing the vasculature/TME, and enhancing the immunogenicity of neoantigens, simultaneously.

Martin et al. proposed the “perpetuation of the TME cancer–immunity cycle” after T cells are primed and expanded when treatment is initiated by adoptive cell therapy (a vaccine carrying an antigen and an adjuvant) preceding the ICD-inducing chemotherapy and/or RT leading to TME normalization, followed by combination with an anti-PD-1 or anti-PD-L1 antibody. Both the efficacy and the safety of each of these treatments can be enhanced by nanomedicines (2). These actions break the vicious cycle of aberrant angiogenesis-induced immunosuppression in tumors with varied phenotypes, leading to the augmentation of angiogenesis by specific immune cells (2).

*Highlight*: The downside of an *in vivo* vaccine is that the neoantigens released by SBRT or chemotherapy will be diluted by the large amount of non-mutant peptides released simultaneously (28), which needs to be overcome using appropriate immune adjuvants.

### 2.9 Phagocytosis and interstitial pressure

For an efficient decrease in interstitial pressure (ISP), two essential elements must be met to lay the foundation for vascular normalization and the cascading immunological effects: one is the increasing number of CCDs, and the second is the prompt and efficient (professional) clearance of the resultant cell debris by phagocytosis. Cumulating necrotic areas would be a good nidus for anoxic cells responsible for therapy resistance and recurrence at a later date. In addition, the dead cells release sustained inflammatory signals, leading to ECM fibrosis (33). DAMPs and nucleotide ATP are known to be passively released from necrotic cells, stimulating a pro-inflammatory response (34), and could also end up with immunosuppression, depending on the context, as discussed elsewhere.

The innate immune system has monocytes, macrophages, and DCs that function as APCs and NK cells. Antibody-dependent phagocytosis (ADCP) is the link between innate and adaptive immunity (35). The most efficient phagocytic cells are M1 and M2 macrophages, the former being distinctly superior in clearing dead cells and debris (36).

The propensity of tumor cell CD47 to bind to signal regulatory protein alpha (SIRPα) on the macrophage, thus creating a checkpoint, spares the tumor cell from phagocytosis. This blockade is overcome by fusion protein or anti-CD47 monoclonal antibodies ((mAbs) or the binding of anti-SIRPα mAbs to SIRPα on macrophages, sensitizing the cells for macrophage phagocytosis. Combining the potency of this along with other checkpoint inhibition on the PD-L1 axis using a biphasic antibody unleashes a potent phagocytic process. Nanoparticle-packaged drugs favoring M1 polarization as foreign bodies are attractive vehicles for intra-macrophage delivery (36). MiR-340 regulates and inversely correlates with the “don’t eat me” signal CD47, and the restoration of the former promotes phagocytosis in pancreatic cancer cells (37). The chemotherapeutic drug paclitaxel induces significant ICD and acts as a universal adjuvant in potentiating ADCP, even enhancing the cetuximab, rituximab, trastuzumab, and anticancer antibodies in cancer cell clearance (38). Cyclophosphamide potentiates the anti-myeloma activity of daratumumab through the latter’s ADCP-dependent cellular phagocytosis specificity (39) and its ability to induce ICD. To overcome the resistance to RT, dual blockade of CD47 and human epidermal growth factor receptor 2 (HER2) concurrently upregulate CD47-mediated augmented anti-phagocytosis (40).

Locally used and systemically tried CpG, an immune adjuvant in activation of adaptive immunity, also overcomes CD47-mediated cancer protective signal by rewiring the metabolic demands for the phagocytosis of macrophages. Studies have shown that CD47-blocking antibodies may be insufficient for bone marrow-derived macrophages (BMDMs) in stimulating phagocytosis. In such situations, CpG-induced antitumor activity was indistinguishable between tumors that expressed or lacked CD47, indicating the presence of additional anti-phagocytic signals (41).

### 2.10 Other cancer cell modifiers

Focusing on increasing CCD throughout ImT is an essential strategy targeting the aberrant vasculature. With every incremental decrease in tumor cell mass, the effectiveness of ImT improves distinctively. The basket contains promising drugs including metabolism inhibitors targeting EC glycolytic metabolism and nanoparticles to miRNAs affecting the VEGF/VEGFR pathway, which is to be screened for prioritization.

One type of cell death is autophagy. It mainly helps in cancer cell survival and is responsible for therapy resistance. However, in certain situations, it helps in tumor cell clearance, resulting in vessel normalization and tumor inhibitory autophagy. Reports of generic drugs such as the first-generation autophagy inhibitor chloroquine (CQ) (5) that had a role to play in *in vivo* normalization through the activation of the Notch signaling pathway, or experimental metronomic therapy/hormone therapy, etc., indicated that exploration is required to identify more specific molecules that can broaden the normalization spectrum without increasing the toxicity.

### 2.11 The integrity of ECM and elimination of dormancy

The structure that supports the vasculature is the ECM; maintaining its suppleness during and even after anticancer therapy is essential to keep the immunological cross-talk preventing a recurrence (25).

Endothelial cells generate a basement membrane predominantly made of specialized thin sheets of ECM made primarily of collagen IV and laminins. In cancer, abnormally thick layers of pericytes cover the basement membrane. A stiff ECM caused by deposition and increased cross-linking of its constituents completes the pivotal components of the hypoxic tumor niche (42). This denser ECM reduces the space for the diffusion of drugs (42).

Both the ECM and its stiffness can activate receptors and mechanosensors, modifying both stromal and malignant cell phenotypes and promoting a spectrum of tumor enhancement with drug resistance. Growth factors such as TGF-β induce collagen/elastin deposition (43). ECM stiffening can cause hypoxia due to increased extracellular pressure. It may reduce the number and function of cytotoxic T cells and, in various studies, negatively correlated with PD-1 blockade therapy, DC migration, and effectiveness (although a study has demonstrated the activation of DCs through the activation of mechano-signal transducers) (43). The degree of stiffness correlates with tumor aggression and poor prognosis as a critical component of the TME. ECM stiffness can physically inhibit immune cell infiltration (44). Stiffness can be evaluated using MRI, CT scan, and elastography (44).

The immune system can confine the cancer cells into long-lasting latency, and fibrosis is known to transition dormant cells to proliferating ones (44). Collagen cross-linking is principally by lysyl oxidase (LOX) contributing to tumor progression, the inhibition of which suppresses fibrosis and increases tumor dormancy (45).

Angiogenic dormancy is a balance of proliferation and apoptosis that does not progress further, but which may reactivate at a later time with perturbations in the TME. There is a question of whether stromal alterations are always reversible or irreversible. The TME of a mouse blastocyst suppressed the tumorigenicity of teratocarcinoma cells and was reprogrammed, resulting in normal chimeric mice. Normal fibroblasts could even reverse the malignant phenotype when chronic inflammation is absent. Embryonic TME can reprogram various cancer cells to a less aggressive phenotype (46).

Eliminating dormancy is the extended effect of combinatorial ImT with a normalized vasculature/TME and a virtuous immunity cycle. This situation mandates that the vasculature retains its integrity, and the TME should be without stiffness for normal immune cell infiltration and cross-talk (immunosurveillance) at the end of all the intensive therapies (42).

Targeting ECM stiffness is an active area of study, which includes using recombinant collagenase to deplete collagen; inhibitors of heat shock protein 47 (Hsp47), LOX, integrin, etc.; and nanoparticles. The accumulation of M1-like TAMs has been observed when a TGF-β inhibitor was combined with nanomedicine. The delivery of nanomedicine improves when the TGF-β receptor is targeted (43). Targeting the TGF-β1/connective tissue growth factor (CTGF) pathway, the chemokine CXCL12, or the chemokine receptor CXCR4 can reduce RT-induced fibrosis and increase T-cell infiltration. Targeting fibroblastic activation protein (FAP) alone has not shown clinical benefit (25). Losartan can deplete dense collagen networks, and TGF-β can be targeted to normalize the ECM. The TGF-β inhibitor tranilast is an approved antihistamine and anti-fibrotic. A combination of the monoclonal antibody DC101, a VEGFR antibody, and an anti-TGF-β1 antibody, apart from normalization, reduced collagen density (42).

## 3 Perspectives

**Figure 2:**
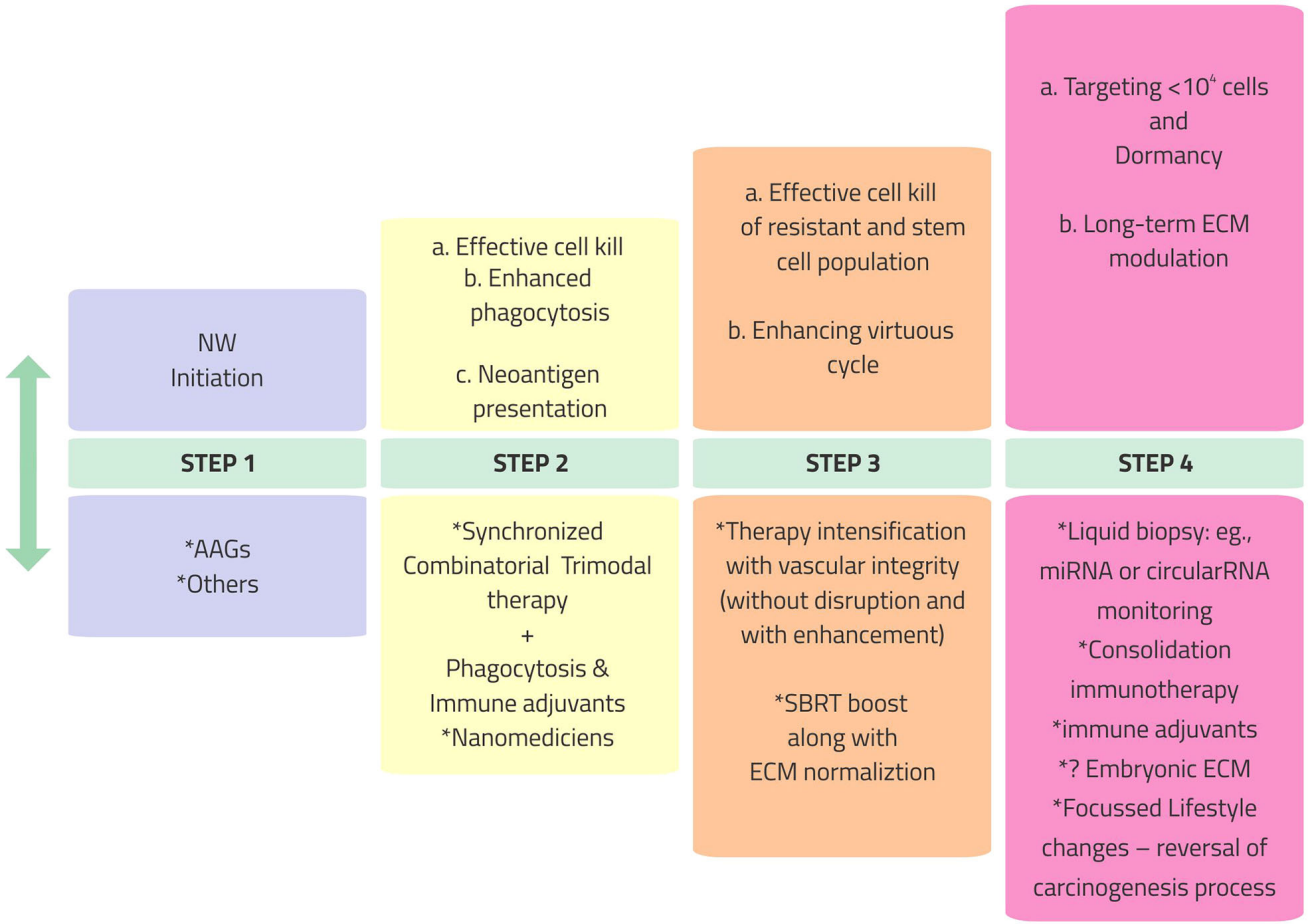
Vascular, immunological, and phenotypic normalization in cancer therapy program: a stepwise resolute approach. *NW*, normalization window; *AAGs*, anti-angiogenics; *SBRT*, stereotactic body radiotherapy; *miRNA*, microRNA; *ECM*, extracellular matrix.

Barring aberrant vasculature, the significant hurdles for immune response cascade for combinatorial ImT are as follows: a) immediate counter-reactive homeostasis response for ICD mitigates the immunological reaction (e.g., the recruitment of MDSCs inhibiting the activity of CD8^+^ T cells and DCs instantly after SBRT); b) generation of massive amounts of nonspecific antigens drowning the cancer cell-specific antigens; and c) the immunosuppressive nature of therapies, especially local RT, when given over a long time (immune exhaustion). These require specific targeting along with the trimodal therapy of AAGs, chemotherapy/RT/SBRT, and ImT, not only in terms of combinations but also in terms of “graceful sequence.” The sequence should glide through four steps (**Figure 2**) of opening the NW as a pre-priming; concurrent effective CCD as the priming step; turning on phagocytosis—antigenicity in step 3, taking care of massive CCD; followed by consolidation ImT with ECM modulation as the fourth step. Opening, enhancing, sustaining, and tracking the NW for synchronization of the available therapies throughout this four-step combinatorial approach is the *desideratum*. The objective of the combination is to harvest the advantages of each conventional treatment with different mechanisms (**Table 1**), simultaneously countering the disadvantages of each with the other.

**Table 1:** Vascular targeting mechanisms, methods, and manifestations.

Mechanisms	Methods (in sequence)	Manifestations
1. Vascular normalization window (pre-priming)	Reversal of aberrant vascular changes by AAGs/nanomedicines Intermittent AAGs	Temporary Extended Intermittent/cyclical
2. Decreased ISP (priming)	Cell killing and priming by RT/CT//AAGs/nanomedicines	Increased perfusion Cancer cell sensitization Increased lymphatic drainage Decreased blood viscosity Increased drug delivery
3. Differential protection of the endothelium and sensitization of cancer cells	Genetic modulation of the endothelium Dual-recombinase technology	Improved cell killing with ICD Neoantigen generation Better immunogenicity
4. Immunological enhancement	ImT	Optimized initiation of immune cascade
5. *In vivo* vaccine generation	Intermittent SBRT Use of immune adjuvants and nanomedicines	A virtuous cycle of CCD/ICD and vascular normalization
6. Enhancing the vasculature	Matched Ang1 accumulation (with Ang2 blockade) for vascular maturation	Prolonged/permanent normalized vasculature HIF1-α disintegration
7. ECM suppleness	Ensuring endothelium, basement membrane, and pericyte integrity Targeting TGF-β1	Immunogenic TME Limited long-term toxicities
8. Combinations of the above	Tracking the vasculature and synchronization	Optimum virtuous cycle of CCD/ICD, neoantigens, and vascular integrity

ISP, interstitial pressure; ECM, extracellular matrix; AAGs, anti-angiogenics; RT, radiotherapy; CT, chemotherapy; ImT, immunotherapy; SBRT, stereotactic body radiotherapy; ICD, immunogenic cell death; CCD, cancer cell death.

### 3.1 Initiation of the NW (step 1)

The complex interactions indicate that intelligent, opportune combinations of existing therapies are required to optimize the vasculature. 1) Presently, the AAG drugs, including the small molecules, are clinically established in several indications and are the best bet to initiate the NW. 2) It is essential to understand the precise combination and timing of Ang2, the angiogenesis initiation molecule, and the accumulation of Ang1 as the vessel maturation mediator along with ImT. 3) Given the short to moderate temporal nature of normalization of the vasculature, “intermittent/cyclical” therapy schedules based on imaging (vasculature-guided therapy) findings are worth exploring. 4) SBRT is an excellent potential immune priming agent for such an intermittent/cyclical approach, within the tolerance of volume, total dose, and possible bleeding toxicities (a matched vascular maturation agent and timed Ang1 administration are worth exploring to overcome bleeding complications). However, since immune resetting by SBRT is instantly countered by cellular survival pathways, adjuvants are required to prevail over the immunosuppressive “recoil.” *The strategy is to overcome the “don’t eat me” CD47 signals by activating* innate immunity mediators receptor-interacting serine/threonine protein kinase 1 (RIPK1) and nuclear factor kappa B (NF-kB) signaling (47).

### 3.2 Optimizing cell killing without endothelial disruption (step 2)

The literature review enumerated above indicates that, for RT to invoke a virtuous cycle when combined with ImT, the following significant conditions must be met: a) the vasculature should be normalized and not disrupted; b) there should be substantial CCD; c) CCD should be predominantly of an immunogenic type (ICD); d) the TME should have the most negligible fibrosis effect to maintain immunological cross-talk; e) the RT schedule should not be prolonged to prevent the immunosuppressive effects of long-term radiation; f) the radiation-treated volume should be limited to avoid significant toxicity; and g) RT should not suppress the immunological effects of the ImT. The RT dose schedule suitable to all these conditions is SBRT, delivered in the dose per fraction range of 5–10 Gy as a boost to the gross disease after standard fractionated RT or to oligometastases, preferably in a cyclical schedule (in divided doses) just preceding each cycle of ImT. The repeated cascades of neoantigen generated, continuing with the flow of mutations, with each “intermittent/cyclical” fraction of SBRT should be converted to a cancer cell-specific target with the concurrent use of immune adjuvants. Neoadjuvant chemotherapy helps to reduce the disease volume and select the chemoresistant population of cells for SBRT and ImT.

### 3.3 Overcoming immunological, hypoxic, and non-delivery in “cold” areas/nodules: Converting limitations of therapies to opportunity

Cancer therapy has always been about appropriate combinations. AAGs, known normalizing agents, albeit for a limited period, are valuable in aiding RT and chemotherapy reach out to immunological cold areas. To an extent, radiation is less active or ineffective against hypoxic compartments, which chemotherapy/ImT can reach. Radiation can reach out to the non-chemotherapy/ImT delivery cold areas of cancer to convert them to hot nodules, especially with modern CCD-inducible SBRT “titrated” dose, by opening up the vasculature. An immunogenic cycle can be created by overcoming the flood of nonspecific antigens through constantly boosting specific neoantigens with adjuvants. Tracking changes in the vasculature with advanced imaging and mRNAs/miRNAs is revolutionary for assessing the extent and timing of the “hotness.” Fast-paced, evolving nanomedicine technology can support all of these for a positive ripple effect.

### *3.4 In vivo* vaccine generation (step 3)

The stratagem of harnessing neoantigen generation using SBRT or chemotherapy in a normalized vasculature environment to interact with the ImT dynamically changing with every bout of mutations would potentially be a game changer. To keep up with the resistant clones and mutations during ImT and combination chemotherapy, SBRT, in divided doses intermittently or cyclically along with ImT, would increase the generation of neoantigen. These neoantigens should be made specific by immune adjuvants or nanomedicines administered concurrently.

### 3.5 Virtuous cycle synchronization (integrating steps 1–3)

**Figure 3:**
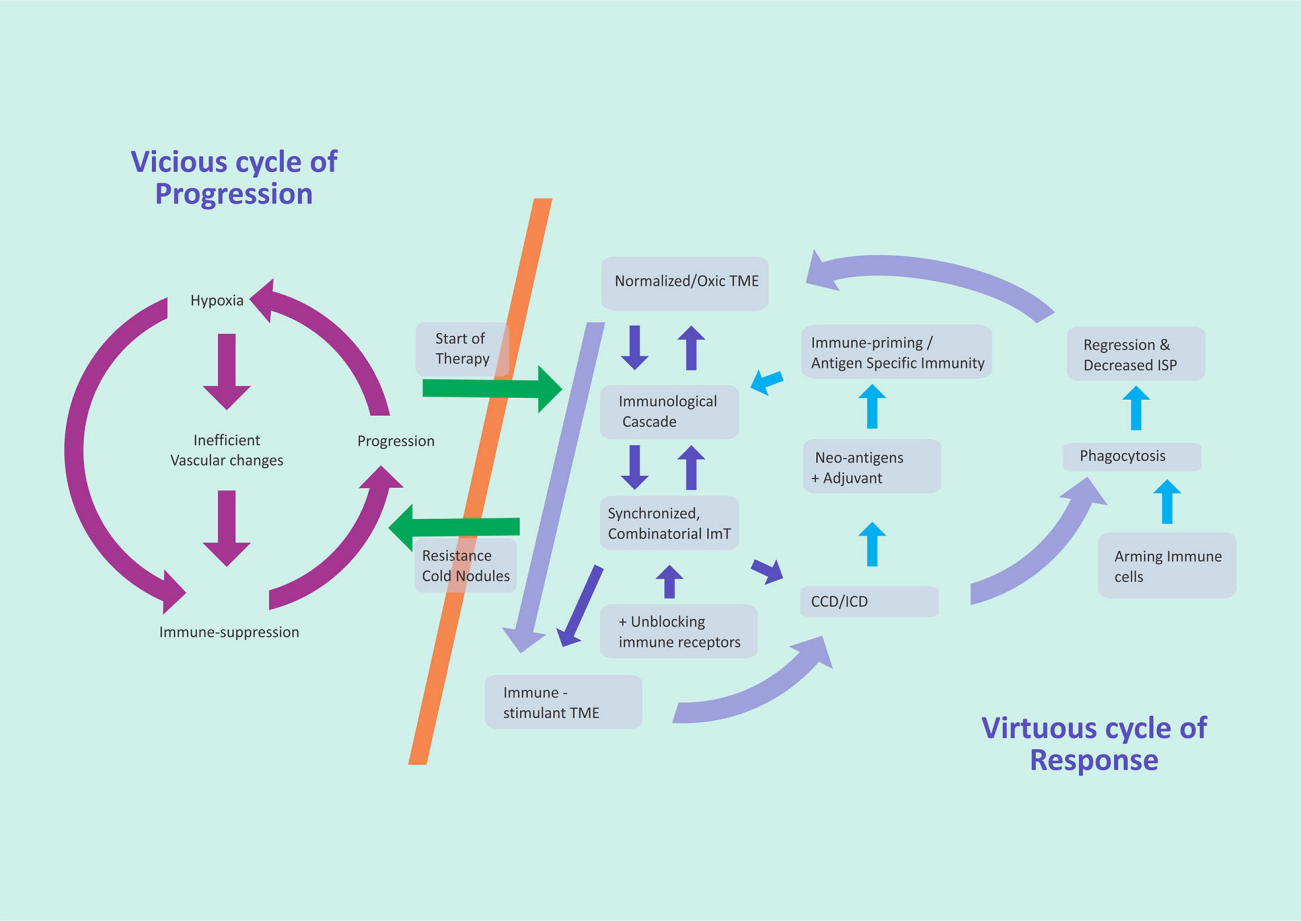
Two cycles that bring out the difference in failure of therapy and cure. It is essential not to take the eye off the normalization of the vasculature during any time of the planned combinatorial immunotherapy. *TME*, tumor microenvironment; *ImT*, immunotherapy; *CCD*, cancer cell death; *ICD*, immunogenic cell death; *ISP*, interstitial pressure.

With the onset of normalization of the vasculature, infiltration of immune cells CD8^+^ and CD4^+^ into the TME increases with the normalization of the vasculature. There is a “reciprocal feedback loop” in which a lymphocyte-admissible TME through vessel normalization has subsequent positive effects on the integrity of the vasculature; the infiltration of CD4^+^ T cells can induce vessel normalization as well (5) (**Figure 3**).

Genetic adoption of cancer cells is ever changing with increasing aggression and is an unfathomable labyrinthine. Therefore, the art and the success of ImT lie in the operative synchronization of vascular normalization with ImT. **Figure 3** displays the ICD causing cancer cell disintegration leading to further cell death, thus setting up the bedrock for a virtuous cycle of *consistent* and long-lasting control of cancer. The other dimension is making optimal use of the phenomenon of T-cell cross-talk and cross-priming (26) in the intricate immunological pathways. NF-kB critically links the inflammatory signals responsible for the proliferation and cytokine generation against anti-pathogen survival response. When the NF-kB pathway is uncoupled from the ICD signal pathway, CD8^+^ T-cell priming is inefficient, even following DAMP cytokine release. Normal cells display “don’t eat me” CD47 signals, which, in tumor cells, need to be targeted for T-cell priming. In an unknown mechanism, the activation of innate immunity mediators takes place. One mechanism could be that RIPK1 and NF-kB signaling interacting within dying cells precipitates vigorous CD8^+^ T-cell cross-priming (47). By activating dendritic cells, CD40 converts cold into hot tumors, and agonistic CD40 antibodies, along with chemotherapy plus ImT, show T-cell-dependent activity. The availability of several CD40 agonists, which possibly act synergistically with STING or TLR agonists, may become the route for reinforced pathways for antigen-presenting DC activation. These approaches may make “a case for priming and not checkpoint” a source of ImT revolution (48).

### 3.6 Modulation of “soil” (step 4)

For seamless normal homeostasis and immunological cross-talk, the ECM should be supple and vascularized. The increasing role of nanomedicines might be the pathway to immune surveillance topped by non-professional phagocytosis, where TME fibroblasts also participate in malignant cell clearance (**Figure 2**).

## 4 Summary

Vessel normalization improves the oxygenation sensitizing the cancer cells for combinatorial therapy, in addition to ImT-induced independent action, and enhances the delivery of ImT drugs to cancer cells. Vascular normalization reverses immunosuppression and enhances the cell adhesion, extravasations, and infiltration of cancer immune cells to create an environment for the finer antigen–antibody reaction for *in situ* vaccination effect, in turn precipitating cancer cell killing, which further improves the normalization of vessels and oxygenation, setting in a virtuous cycle for cancer elimination (**Figure 3**). Increasing the cancer cell killing by apoptosis on the one hand and simultaneously improving the ICD initiated by the normal immune cells of the body with the presence of adequate oxygen (*via* normalized vasculature) on the other hand is the optimum approach with ImT, which also keeps toxicity to the minimum.

The success of ImT, even with cold nodules, lies somewhere in the middle of the synchronization of the 11 interdependent points of vascular targeting discussed above. The situation is what Robert Frost quotes: “We dance round in a ring and suppose, but the secret sits in the middle and knows.”

## Author contributions

The author confirms being the sole contributor of this work and has approved it for publication.

## Acknowledgments

The author thanks Ramesh BS, Asst. Manager–Graphic Designer, Aster CMI, Bangalore, India, for creating the figures in this article.

## Conflict of interest

The author declares that the research was conducted in the absence of any commercial or financial relationships that could be construed as a potential conflict of interest.

## Publisher’s note

All claims expressed in this article are solely those of the authors and do not necessarily represent those of their affiliated organizations, or those of the publisher, the editors and the reviewers. Any product that may be evaluated in this article, or claim that may be made by its manufacturer, is not guaranteed or endorsed by the publisher.
